# Evaluation of four different standard addition approaches with respect to trueness and precision

**DOI:** 10.1007/s00216-024-05725-8

**Published:** 2025-01-10

**Authors:** Gerhard Gössler, Vera Hofer, Walter Goessler

**Affiliations:** 1 Institute of Chemistry, Analytical Chemistry, University of Graz, Graz, Austria; 2 Institute of Operations and Information Systems, University of Graz, Graz, Austria

**Keywords:** Standard addition, Trueness and precision, Bias and variability, Extrapolation, Interpolation, Normalization, Inverse regression, Approximation formulas

## Abstract

**Abstract:**

This work provides a statistical analysis of four different approaches suggested in the literature for the estimation of an unknown concentration based on data collected using the standard addition method. These approaches are the conventional extrapolation approach, the interpolation approach, inverse regression, and the normalization approach. These methods are compared under the assumption that the measurement errors are normally distributed and homoscedastic. Comparison is done with respect to the two most important characteristics of every estimator, namely trueness (bias) and precision (variability). In addition, the authors supply, if not already available, mathematical formulas to approximate both quantities. Also, a real-world data set is used to illustrate the performance of all four methods. It turns out, that, given that all assumptions underlying the use of the standard addition method apply, the common extrapolation method is still the most recommendable method with respect to bias and variability. Nonetheless, if additional concerns come into play, other methods like, for example, the normalization approach in the case of increased problems with outliers might also be of interest for the practitioner.

**Graphical abstract:**

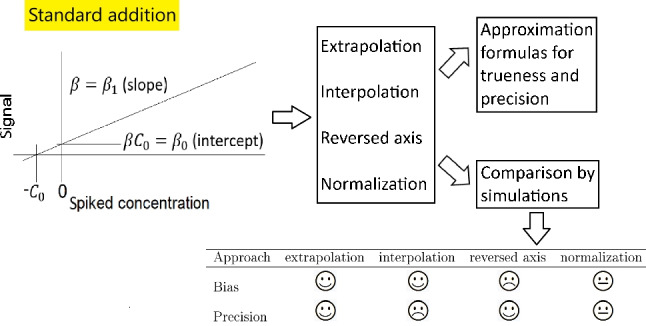

**Supplementary Information:**

The online version contains supplementary material available at 10.1007/s00216-024-05725-8.

## Introduction

In this work, four approaches proposed for the evaluation of standard addition results are statistically scrutinized. These approaches are the well-established extrapolation approach and three alternative approaches, which are intended to improve the quality of the outcome of standard addition in terms of the trueness and precision of the estimator and/or the simplicity of the statistical analysis of the results. All of these approaches were evaluated in detail on the basis of extensive simulation studies and—primarily for illustrative purposes—application to a real data set. The supplementary material contains additional mathematical considerations, namely the derivation of some of the approximation formulas for bias and variance of the different estimators for the unknown concentration.

**Fig. 1:**
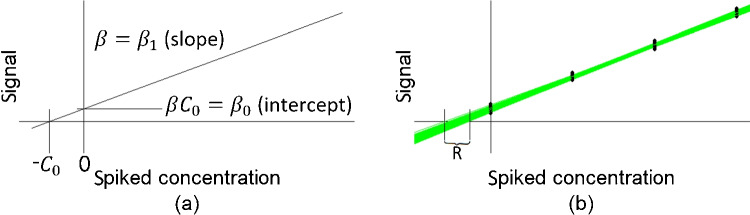
**a** Basic principle of standard addition. Without error, the unknown concentration $C_0$ can be determined exactly by computing $\beta _0/\beta _1$. **b** Due to measurement error, the measurements (within the ranges given by the black vertical bars) and therefore also the estimated regression lines (green lines) show considerable variation when the process of standard addition is repeatedly and independently applied. The possible estimates $\hat{C}_0$ are therefore scattered over the range indicated by *R*

To recap, the well-known standard addition method is intended as a remedy in cases in which a matrix has a “rotational” but no “translational” effect [1] (the matrix affects only the slope of the calibration function but not its intercept) and, due to a lack of pure matrix, no external calibration can be performed. The essential assumptions of this approach are that the relationship between analyte concentration and measurement signal is linear and that the blank value is not significantly different from zero. If these assumptions are fulfilled, the relationship between measurement signal *y* (response) and concentration *C* (independent variable, denoted *x* for spiked concentrations in the following) can, in an ideal setting where also no measurement error is present, be stated as follows (see Fig. 1a):

$$\begin{aligned} y=y(C)=\beta C. \end{aligned}$$

Therefore, if an unknown concentration $C_0$ is present in a sample, the observed signal $y(C_0)=\beta C_0$. When applying standard addition, the sample is subsequently spiked with additional amounts of the analyte such that the observed signal after spiking, $y(C_0+x)$, equals

$$\begin{aligned} \beta (C_0+x) = \beta C_0 + \beta x =: \beta _0 + \beta _1x. \end{aligned}$$

Therefore, the unknown concentration $C_0$ equals

$$\begin{aligned} \frac{y(C_0)}{\beta }=\frac{\beta C_0}{\beta }=\frac{\beta _0}{\beta _1} \end{aligned}$$

which results in the so-called extrapolation approach.

Unfortunately, even if the measurement system is unbiased, the measurements are overlain with measurement error, i.e., when assuming normally distributed errors

1 $$\begin{aligned} Y(C_0 + x)=\beta _0+\beta _1x+\varepsilon , \quad \varepsilon \sim N(0,\sigma ). \end{aligned}$$

Throughout this work, the errors are considered to be homoscedastic, i.e., $\sigma$ does not depend on the measured concentrations.

These errors have the consequence, that only the estimates $\hat{\beta }_0$ and $\hat{\beta }_1$ of the true parameters $\beta _0$ and $\beta _1$ are available, which are, though still unbiased, themselves subject to error (see Fig. 1b). Depending on the concentrations at which the measurements are taken (the experimental design) and the extent of the measurement error, these parameter estimates are distributed as follows:

$$\begin{aligned} \hat{\beta }_0 \sim N \left( \beta _0 ,\sigma \left( \frac{\sum _{i=1}^{n} x_i^2}{n\sum _{i=1}^{n} (x_i - \overline{x})^2} \right) ^{\frac{1}{2}} \right) \quad , \quad \hat{\beta }_1 \sim N \left( \beta _1 ,\sigma \left( \frac{1}{\sum _{i=1}^{n} (x_i - \overline{x})^2} \right) ^{\frac{1}{2}} \right) \end{aligned}$$

with $x_i,i=1,...,n,$ denoting the values of the independent variable.

Due to this variability, also, the estimator $\hat{C}_0$ of the unknown concentration comes now with some amount of uncertainty, since it is the quotient of two random variables: $\hat{C}_0=\hat{\beta }_0/\hat{\beta }_1$.

What is of course interesting now is the distribution of $\hat{C}_0$ which is, due to our assumptions, the quotient of two normally distributed quantities. This question can be solved only approximately, and there are several approaches that can be applied to approximate bias and variance of $\hat{C}_0$. [2] for example present two approaches which come to the same result with respect to the variability of the estimator of the extrapolation method: firstly, the method of propagation of error [3], which works well also in all other cases if applied properly. Problems arise if, for example, the correlation between intercept and slope is not correctly taken into account which would have the consequence that the variance of the estimator would be underestimated (an error that can, according to [2], even be found in textbooks). A second approach to determine the variance of $\hat{C}_0$ is the “extrapolation method” which is based on the so-called “inverse regression problem” (see [4, 5]). In the respective literature, also, the delta method [6] is mentioned, e.g., [7], which is derived from the method of propagation of error to obtain the asymptotic distribution of a random variable, but in both cases, the approximation of mean and variance of a random variable which is given as a function of random variables with known means and variances is found by applying Taylor series expansion. The general application of the Taylor series expansion for the approximate determination of mean and variance can, for example, be found in [8] p.161ff. Therefore, depending on the approach and the quantity in question (i.e., bias or variance), Taylor series expansion is applied to $\hat{C}_0$ either as a function of $\hat{\beta }_0,\hat{\beta }_1$ or as a function of $Y_1,...,Y_n$, which in particular means that $\hat{C}_0$ is either differentiated with respect to $\hat{\beta }_0,\hat{\beta }_1$ or $Y_1,...,Y_n$. For the extrapolation approach, the variance of $\hat{C}_0, \sigma _{\hat{C}_0}^2$, is now approximately given by the following:

2 $$\begin{aligned} \sigma _{\hat{C}_0}^2 \approx \left( \left( \frac{\sigma _{\hat{\beta }_0}}{\beta _0}\right) ^2 + \left( \frac{\sigma _{\hat{\beta }_1}}{\beta _1}\right) ^2 - 2\rho _{\hat{\beta }_0\hat{\beta }_1}\left( \frac{\sigma _{\hat{\beta }_0}}{\beta _0}\right) \left( \frac{\sigma _{\hat{\beta }_1}}{\beta _1} \right) \right) C^2_0 \end{aligned}$$

with $\rho _{\hat{\beta }_0\hat{\beta }_1}$ denoting the correlation $\rho$ of $\hat{\beta }_0$ and $\hat{\beta }_1$ which is given by

$$ \rho =\rho _{\hat{\beta }_0\hat{\beta }_1}=\frac{-\sum _{i=1}^{n} x_i}{\sqrt{n\sum _{i=1}^{n} x_i^2}}. $$

By plugging in the estimators for all unknown quantities, one gets an estimator for $\sigma _{\hat{C}_0}^2$ based on measured data:

$$\begin{aligned} s_{\hat{C}_0}^2 = \left( \left( \frac{s_{\hat{\beta }_0}}{\hat{\beta }_0}\right) ^2 + \left( \frac{s_{\hat{\beta }_1}}{\hat{\beta }_1}\right) ^2 - 2\rho _{\hat{\beta }_0\hat{\beta }_1}\left( \frac{s_{\hat{\beta }_0}}{\hat{\beta }_0}\right) \left( \frac{s_{\hat{\beta }_1}}{\hat{\beta }_1} \right) \right) \hat{C}^2_0. \end{aligned}$$

This approximation works pretty well if the ratio $\beta _1/\sigma$ is above a certain limit, i.e., it is good as long as it is very unlikely, that the denominator takes on values close to zero. Therefore, as a rule of thumb, we suggest $\beta _1/(\sigma (\sum _{i=1}^{n} (x_i - \overline{x})^2)^{-0.5})>12$, but also different ratios are reported in literature [9]. Especially if this rule of thumb is observed, the distribution of $\hat{C}_0$ is considered to be approximately normal. Therefore, the formula for $\sigma _{\hat{C}_0}^2$ cannot only be used for judging the performance of the estimator but also for constructing a confidence interval (CI) for $C_0$. For more information on the distribution of the ratio of two normally distributed random variables, the interested reader is referred to [10, 11] and [12].

The variability of the estimator for $C_0$ derived by the extrapolation approach as approximated by formula 2 gave rise to the interpolation approach in an attempt to reduce the estimator variability. Further efforts to improve the handling and robustness of the extrapolation estimator resulted in reversed regression and the normalization approach. As to our knowledge, there is no thorough comparison of these different approaches available in literature regarding the goodness of the respective estimator as expressed by trueness and precision. We bridge this gap by deriving approximation formulas for these quantities and providing the results of simulation studies.

In order to facilitate the understanding of the different approaches this work is hereinafter organized as follows: The “Methods” section presents all four approaches to be compared in this work. One of the main aims of the “Methods” section is to present all approaches in a unified notation, as many different notations can be found in the literature. Although all these notations are equivalent, we have chosen the standard notation used in regression analysis, which—at least in our opinion—promotes understanding of the topic in general and also with regard to the details in which the approaches differ. The “Results” section provides the approximation formulas and shows the application of the four approaches and the respective approximation formulas to the data set given by [13]. In addition, some simulation results combined with the corresponding results gained by applying the approximation formulas are provided. These results are discussed in detail in the “Discussion” section, highlighting in particular the differences between the approaches with respect to bias and variance. The “Summary” section contains a summary, and last but not least, the derivation of the approximation formulas can be found in the supplementary material.

## Methods

**Fig. 2:**
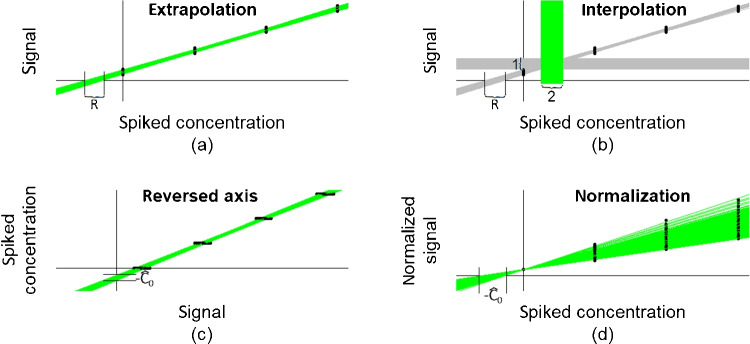
Graphical comparison of the different standard addition approaches

The extrapolation approach, as described in the “Introduction” section, estimates the unknown concentration $C_0$ as $\hat{C}_0^e=\hat{\beta }_0/\hat{\beta }_1$, with $\hat{\beta }_0$ and $\hat{\beta }_1$ denoting the estimates for intercept and slope gained by simple linear regression applied to the data generated by standard addition. Graphically, the intersection of the regression line and the *x*-axis is equal to $-\hat{C}_0^e$. This is illustrated in Fig. 2a. Due to the measurement error, repeated application of standard addition yields differing regression lines. In Fig. 2, 1000 such regression lines are depicted as green (a, c, and d) or gray (b) lines. Due to this variability, also, the resulting estimates vary, which is indicated by the respective ranges. In the case of the extrapolation approach, the corresponding range is denoted as *R*.

The interpolation approach is intended to improve the precision of the estimate, i.e., to supply an estimator $\hat{C}_0^i$ with reduced variance compared to $\hat{C}_0^e$. The idea is as follows ([14, 15] and [16]): The estimation is shifted to the region covered by spiking by calculating

$$\begin{aligned} \hat{C}_{0}^{i} = (2 y_{1} - \hat{\beta }_{0})/\hat{\beta }_{1}, \end{aligned}$$

with $y_{1}$ denoting the observed (averaged) signal for the unspiked sample, to obtain a concentration estimator with lower variance due to the lower variance of the regression line within the range of the available data compared to the variance of the regression line outside of this range—hence the designation “interpolation.” Graphically (see Fig. 2b), $\hat{C}_0^i$ is given by the *x*-value (curly bracket 2) of the intersection of the random horizontal line ($y=2y_{1}$, curly bracket 1) and the random regression line ($y=\hat{\beta }_0 + \hat{\beta }_1x$, curly bracket *R*). Contrary to the basic idea of the interpolation approach, the range indicated by curly bracket *R* ($\hat{C}_0^e$) is narrower than the range indicated by curly bracket 2 ($\hat{C}_0^i$).

The reversed-axis approach ([7, 13]) is intended to reduce the effort for the estimation of the precision, i.e., of $s_{\hat{C}_0}$. This is done by reversing the role of the analyte concentration and the instrument response in linear regression. The former serves now as the dependent variable and the latter as the independent variable. Therefore, by rearranging the assumed relationship

$$\begin{aligned} y=\beta _0 + \beta _1x + \varepsilon { to}x=- \beta _0/ \beta _1 + y/\beta _1 + \varepsilon /\beta _1=:\beta ^r_0 + \beta ^r_1y + \varepsilon _r \end{aligned}$$

it can be seen that the estimator $\hat{\beta }^r_0$ of $\beta ^r_0$ is the negative value of the desired estimate for $C_0$, i.e.,

$$\begin{aligned} \hat{C}^r_0= -\hat{\beta }^r_0. \end{aligned}$$

The variance of $\hat{\beta }^r_0$ can be easily estimated by applying the formula for the variance of the estimator of the y-intercept of simple linear regression to the reversed data, i.e.,

$s^2_{\hat{C}^r_0} = s_r^2\frac{\sum _{i=1}^{n} y_i^2}{n\sum _{i=1}^{n} (y_i - \overline{y})^2}$ with $s_r^2$ denoting the estimate of the variance of $\varepsilon _r$.

Graphically (see Fig. 2c), $-\hat{C}^r_0$ is given by the intersection of the regression line and the *y*-axis. Due to the measurement error, the negative estimate can be found within the range bounded by the two vertical lines below the *x*-axis (due to different axis scaling, this range cannot be directly compared to the other estimator ranges indicated in Fig. 2).

The last approach considered is the so-called normalization approach [16] which is intended to mitigate the effects of outliers. The idea is to normalize all observations $y_i, i=1,...,n_e$, with $n_e$ denoting the number of different spiked concentrations, with respect to $y_{1}$, the observation for the unspiked sample. Normalization is carried out by dividing all observations by $y_{1}$, i.e. $y^n_i= y_i/y_{1}$. Therefore, it follows from $y=\beta _0 + \beta _1x + \varepsilon$ that

$$\begin{aligned} y^{n}_{i}=\beta _{0}/y_{1} + (\beta _{1}/y_{1})x_{i} + \varepsilon /y_{1} =: \beta ^{n}_{0} + \beta ^n_1x_i + \varepsilon ^n_i. \end{aligned}$$

If more than one series is measured, each series has to be normalized with respect to its unspiked measurement. It has to be pointed out that normalization has two important consequences:

Firstly, all normalized observations for the unspiked samples are equal to 1 which means, that in the subsequent regression analysis, the regression line has to be forced through 1 which is equal to a fixed *y*-intercept, i.e., $\hat{ \beta }^n_0$ is always equal to 1. Therefore, the estimator $\hat{C}^n_0$ for the unknown concentration $C_0$ is now given by

$$\begin{aligned} \hat{C}^n_0 = 1/\hat{\beta }^n_1 \end{aligned}$$

since $\beta _0/\beta _1 = (\beta _0/y_{1})/(\beta _1/y_{1})=\beta ^n_0/\beta ^n_1$ and $\hat{\beta }^n_0 = 1$. This result is also reached by the following consideration: Since $y_{1} \sim N(\beta _0,\sigma )$, the expectation of $\hat{\beta }^n_1$ is approximately $\beta _1/\beta _0=1/C_0$ (there will be some bias), i.e., $\hat{C}^n_0 = 1/\hat{\beta }^n_1$. Graphically (see Fig. 2d), the intersection of the regression line and the *x*-axis is equal to $-\hat{C}_0^n$, which can, due to the variability of the measurements, be found in the range bounded by the two vertical lines on the left-hand side of the *y*-axis. The figure also shows the fixed intercept which is always equal to 1 and the heteroscedasticity brought about by normalization.

**Table 1:** Approximation formulas for the bias of $\hat{C}_0$, bias$=E(\hat{C}_0)-C_0$

Approach	Bias
Extrapolation$^a$	$\sigma ^2\frac{S_{xx}}{S_{xy}^2}(C_0+\overline{x})$
Interpolation$^b$	$\sigma ^2\frac{S_{xx}}{S_{xy}^2}(C_0+\overline{x})$ or, alternatively
	$$\frac{\sigma ^2n_rS_{xx}}{ S_{xy}^3}\left( \overline{x}((2/n_r-1/n)S_{xy} + \overline{x}(\beta _0-\beta _1\overline{x})) + (\beta _0-\beta _1\overline{x})S_{xx}/n_r \right) $$
Reversed axis$^c$	$-\frac{\overline{y}(n-3)\sigma ^2}{\beta _1S_{yy}}$
Normalization$^d$	$\frac{n_r\sigma ^2\beta _0}{\beta _1^3\left( \sum \limits _{i=1}^n x_i^2 \right) ^2}\left( \frac{\left( \sum \limits _{i=1}^{n_e}(y_{1i0}x_i)\right) ^2}{\beta _0^2}-\frac{\beta _1\sum \limits _{i=1}^n x_i^2 \sum \limits _{i=1}^{n_e}(y_{1i0}x_i)}{\beta _0^2} + \sum \limits _{i=1}^{n_e} x_i^2 \right)$
$^{a, b, d}$...derived by authors (see supplementary material), $^c$...[7]	

Secondly, the quotient of random variables is a random variable whose variance depends not only on the variance, but also on the expectation of the random variables involved in the quotient [12]. The latter has the consequence that the error term is now heteroscedastic (see Fig. 2d) in a way that makes a proper estimation of $\sigma$, the variance of the measurement error, based on the regression with respect to the normalized data impossible. Therefore, also, the variance of $\hat{\beta }^n_1$ and thus the variance of $\hat{C}^n_0, \sigma ^2_{\hat{C}^n_0}$, cannot be determined easily. The respective approach according to the supplementary material of [16] therefore estimates the variance of the slope estimator, $\sigma ^2_{\hat{\beta }_1^n}$, not by applying an approximation formula (like that given in Table 2), but alternatively by calculating the slope for each of the independently taken series of measurements separately. This set of different estimates for $\beta _1^n$ is now used to estimate the variability of $\hat{\beta }_1^n$. Let us denote the number of the different series measured by $n_r$, $n_r\ge 1$, and obviously, to make this approach work, $n_r$ needs to be greater than 1. In order to remain consistent in the simulations, we adopted this approach and also used this estimate for $\sigma ^2_{\hat{\beta }_1^n}$, $s^2_{\hat{\beta }_1^n}$, subsequently to estimate $\sigma$ by rearranging the approximation formula for $\sigma ^2_{\hat{\beta }_1^n}$. This estimate for $\sigma$ can then be used to calculate an estimate for the bias by applying the respective approximation formula in Table 1. Unfortunately, this approach overestimates $\sigma _{\hat{\beta }_1^n}$ on average by a factor of roughly $\sqrt{n_r-1}$. This can be regarded as similar to the relationship between the standard deviation of a random variable *X* with variance $\sigma _X^2$ and the standard deviation of the corresponding sample mean $\overline{X}_n$ based on *n* observations for which $\sigma _{\overline{X}_n} = \sigma _X/\sqrt{n}$. Therefore, correcting $s_{\hat{\beta }_1^n}$ by multiplying it with a factor of around $1/\sqrt{n_r-1}$ is necessary to avoid overestimation which subsequently also leads to a correction of $s_{\hat{C}^n_0}$ by the same factor. A further problem is to determine the proper degrees of freedom, *df*, for calculating a reasonable confidence interval for $C_0$. Simulations indicate that especially the number of different spiked concentrations, $n_e$, seems to have a rather neglectable influence on the adequate choice of *df*. For example, simulations indicate that $\hat{C}^n_0 \pm \frac{s_{\hat{C}_0^n}}{\sqrt{n_r-1}}t_{df, 0.975}$ yields a 95% CI for $C_0$, when choosing *df* around 4 (depending on $n_r$) irrespective of $n_e$. However, in our opinion, it would be necessary for applying this correction in practice, to base this choice for *df* on a solid theoretical argument that needs to be further investigated and is not yet available. However, overestimation of $\sigma ^2_{\hat{\beta }_1^n}$ obviously has the consequence, that the bias is on average overestimated as well as the variance of $\hat{C}_0$. The latter yields confidence intervals which are, on average, too wide and therefore exceed the chosen confidence level. If the estimate for $\sigma$ gained by regression analysis applied to the unnormalized data is used to calculate bias, variance and the CI for the normalization approach the results are in line with the simulation results and the chosen confidence level (using this approach $df=n-2$), but in our opinion, it would be inconsistent to extend the normalization approach by an additional analysis of the unnormalized data.

As also pointed out in [16], when applying the normalization method, proper measurement of the unspiked sample is crucial. Outliers in the case of the unspiked measurement would severely worsen the result of the normalization method compared to the extrapolation method, but if outliers occur with respect to spiked samples that cannot be removed before regression analysis, it can outperform the extrapolation approach. That the unaffectedness of the unspiked samples is of outermost importance for the normalization approach has its reason in the fact that the gain in robustness with respect to outliers is achieved by fixing the *y*-intercept after normalization ($\hat{ \beta }^n_0=1$). Therefore, it is essential to be able to rely on the measured values for the unspiked samples.

Before discussing the performance of the different approaches in more detail in the next section, we want to point out the following important facts to avoid misunderstandings:

1. The quotient of two random variables has the following property: Since, in general, for a random variable *X*, the expectation of its reciprocal value *E*(1/*X*) is not equal to the reciprocal value of its expectation, i.e., 1/*E*(*X*), we have that the estimator for $\hat{C}_0=\hat{\beta }_0/\hat{\beta }_1$ is biased, i.e., $E(\hat{C}_0) \ne E(\hat{\beta }_0)/E(\hat{\beta }_0) = \beta _0/\beta _1$. This means that the estimator for the unknown concentration is on average not identical to the true value, but more or less off [17].
2. The chosen number of measurements and spiked concentrations (i.e., the *x*-values ) represent the *design* of the standard addition approach. The design has a significant impact on the variance (i.e., precision) of the estimator for the unknown concentration [18]. Therefore, comparing different approaches is only reasonable if the designs do not differ.
3. Looking only at the confidence band for the regression line can be misleading, as the interpolation method will show. With this method, the estimate is determined by the intersection of the regression line with a horizontal line which is not fixed (as the *x*- or *y*-axis), but is itself subject to chance.

## Results

As already mentioned, bias and variance are the most important performance criteria of a statistical estimator. Both quantities should of course be as small as possible, although there might be some trade-off in the sense that a larger bias might be acceptable when it comes along with a correspondingly smaller variance.

The aim of this work is to deliver results based on theoretical considerations and extensive simulations. One of the goals is to provide approximation formulas for bias and variance as these formulas cannot only be used to better understand the behavior of the different approaches and to judge their performance, but are also useful in practice, i.e., to calculate confidence intervals for the unknown concentration $C_0$. To the best of our knowledge, our publication contains for the first time a complete set of formulas for all four investigated standard addition approaches for both bias and variance which are ready to use for the practitioner since all necessary components are known/can be estimated using linear regression. In addition, a standardized and widely used notation is applied to improve the accessibility of the formulas.

Besides the use of formulas, bias and variance of the estimator can be determined by applying Monte Carlo methods, in the following denoted simulations. Such simulations utilize the generation of a huge number of synthetic random samples to yield numerical results (see [19]). They do not rely on the approximation formulas, but are based solely on the relationship given in formula (1) to randomly generate observations (“measurements”) used for calculating estimates and CIs for $C_0$ for all four approaches in parallel. By iterating this process *t* times, *t* random estimates and CIs for each approach are generated which can subsequently be used to calculate bias and variance of $\hat{C}_0^x, x=e,i,r,n,$ and coverage probabilities and average widths of the respective CIs. These simulation results cannot only be used to judge and compare the performance of the several approaches, but can also be used to validate the approximation formulas since the results of simulation and proper approximation have to be very close.

We would like to point out that although [16] analyze the same standard addition approaches for bias and variance, there are significant differences between our work and theirs. In contrast to our work, [16]do not provide formulas for the bias and the formulas provided for the variances in the case of the interpolation (Eq. (7)) and the normalization approach (Eq. (12)) contain quantities which are not easy to compute. In particular, these quantities are the following: in Eq. (7), it is $S_{yb}, S_{ym}$, and $S_{bm}$, and in Eq. (12), it is $S_{m_1 }$, which would have to be stated explicitly so that Eq. (7) and Eq. (12) can be applied without further elaboration. [16] leave it open how they are to be calculated. In addition, we based our simulations on the usually applied basic assumptions of standard addition (homoscedastic and normally distributed errors, blank value not significantly different from zero, linearity). Due to these standardized conditions, deviations from the basic assumptions are excluded which allows a clear statement to be made. In contrast to our approach, real-world data are often overlain by the characteristics of the different instrumental methods used which can obscure the underlying relationship. If discrepancies between experiment and theory occur, this indicates that additional factors play a role that lead to deviations from the basic assumptions. This is also argued by [16], who are extensively discussing the potential influences of the different instrumental techniques on the outcome of their comparisons. However, neither [16] nor this work makes the other work superfluous, as theory and experiment are always complementary methods of investigation, both of which are indispensable. This means that theory must be tested with experiments and, conversely, theoretical considerations are necessary to understand and model the observations from the experiments and make them accessible for general application. The discrepancies between experiment and theory should provide the impetus for further research and improvement of the method (e.g., applying weighted regression in the case of heteroscedasticity).

In the following, consider that $$n_r$$ different series are measured and that each series *r* consists of $$n_e$$ single observations $$Y_{ri}, r=1,...,n_r, i=1,...,n_e$$, i.e., the total number of observations $$n=n_r n_e$$. Therefore, the used spiked concentrations in vector notation are $$\varvec{x}=(x_{11},...,x_{1n_e},x_{21},...,x_{2n_e},x_{31},...,x_{n_rn_e})$$, and the vector of the measured responses is $$\varvec{Y}=(Y_{11},...,Y_{1n_e},Y_{21},...,Y_{2n_e}, $$ $$ Y_{31},...,Y_{n_rn_e})$$. Keep in mind that $$x_{r1}=0$$ for all $$r=1,...,n_r$$ and that for all $$i=1,...,n_e \ x_{ji}=x_{li}$$ for all $$j,l = 1,...,n_r$$.

**Table 2:** Approximation formulas for $\sigma ^2_{\hat{C}_0}$, the variance of the estimator $\hat{C}_0$

Approach	Variance
Extrapolation$^a$	$\left( \left( \frac{\sigma _{\hat{\beta }_0}}{\beta _0}\right) ^2 + \left( \frac{\sigma _{\hat{\beta }_1}}{\beta _1}\right) ^2 - 2\rho _{\hat{\beta }_0\hat{\beta }_1}\left( \frac{\sigma _{\hat{\beta }_0}}{\beta _0}\right) \left( \frac{\sigma _{\hat{\beta }_1}}{\beta _1}\right) \right) C^2_0$
Interpolation$^b$	$\left( \left( \frac{\sigma _{\hat{\beta }^i_0}}{\beta _0}\right) ^2 + \left( \frac{\sigma _{\hat{\beta }_1}}{\beta _1}\right) ^2 - 2\rho _{\hat{\beta }_0\hat{\beta }_1}\left( \frac{\sigma _{\hat{\beta }_0}}{\beta _0}\right) \left( \frac{\sigma _{\hat{\beta }_1}}{\beta _1}\right) \right) C^2_0$
	with $\sigma ^2_{\hat{\beta }^i_0} = \sigma ^2_{\hat{\beta }_0} + 4\sigma ^2\left( \frac{1}{n_r} - \frac{1}{n} - \frac{\overline{x}^2}{ns_x^2} \right)$
Reversed axis$^{c, d}$	$\sigma ^2/\beta _1^2\frac{\sum \limits _{i=1}^{n} y_i^2}{nS_{yy}} \quad$ or $\quad \sigma ^2/\beta _1^2\left( \frac{1}{n} + \frac{\overline{y}^2S_{xx}}{S^2_{xy}} \right)$
Normalization$^e$	$\frac{C_0^2n_r\sigma ^2}{\left( \beta _1 \sum \limits _{i=1}^{n} x_i^2\right) ^2}\left( \left( \frac{1}{\beta _0} \sum \limits _{i=1}^{n_e} y_{1i0}x_i \right) ^2 + \sum \limits _{i=1}^{n_e} x_i^2\right) =C_0^4\sigma ^2_{\hat{\beta }_{1 norm}}$
$^{a,c}$...various sources, $^{b, e}$...derived by authors (see supplementary material), $^d$...[7]	

### Approximation formulas

Since no closed forms for bias and variance of the estimators for $C_0$ exist, we need to resort to approximation formulas to enable the calculation of approximate values for these quantities. To derive these formulas, let the errors be normally distributed and homoscedastic and $y_{ri0}:=\beta _0 + \beta _1x_{ri}$ denote the expected value of a measurement given spiked concentration $x_{ri}$. Furthermore, define

$$ S_{xx}:=\sum _{i=1}^n (x_i - \overline{x})^2, \ S_{yy}:=\sum _{i=1}^n (y_i - \overline{y})^2, \ S_{xy}:=\sum _{i=1}^n (x_i - \overline{x})(y_i - \overline{y}). $$

By making use of Taylor expansions (propagation of error), we get the approximation formulas for bias and variance of the different estimators for the unknown concentration that can be found in Tables 1 and 2.

Some of these results can already be found in literature or have, where not available, been derived by the authors. The derivation can be found in the supplementary material.

Note that these formulas contain the true parameters of the underlying relationship, e.g., $\beta _0$ is the true but normally unknown *y*-intercept, and $\sigma ^2_{\hat{\beta }_0}$ is the true variance of its estimator. Of course, the true parameters are known in theoretical considerations and simulations, but when these formulas are used in practical applications, these unknown parameters have to be replaced by proper estimates.

Keep in mind that a thorough mathematical analysis with respect to the evaluation of the goodness of the approximation formulas as well as of the performance of the CIs based on these formulas would be extremely difficult or perhaps even impossible. Also, a respective evaluation based on just one dataset is not possible. Therefore, extensive simulations have been employed to investigate the performance of the approximation formulas and of the respective CIs. These simulations have been performed by utilizing the programming language  [20] (R version 4.2.3) which has also been used to create all figures shown in this work. There has been good agreement between the results gained by the simulations and the approximation formulas indicating the validity of the derived formulas.

**Fig. 3:**
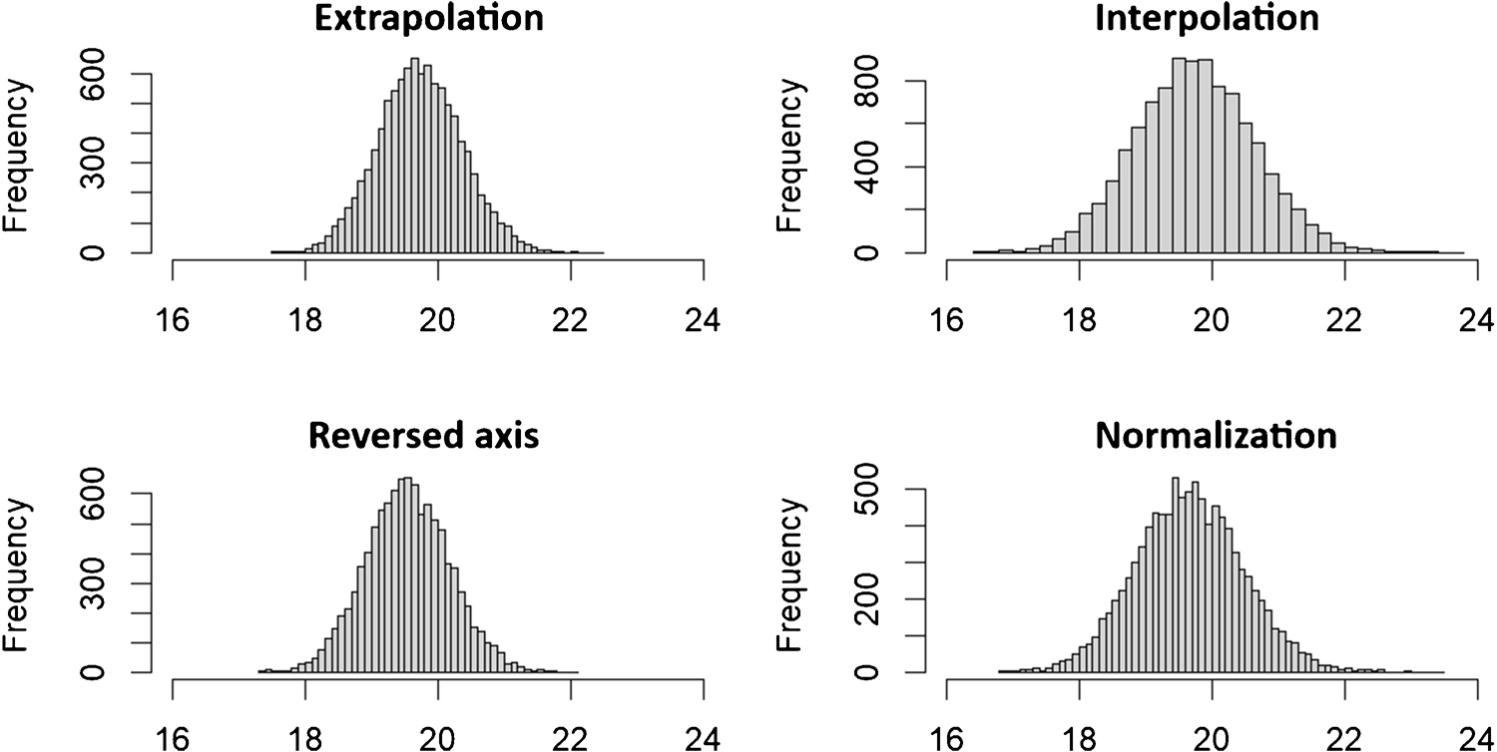
Histograms showing $10^4$ simulated estimates for $C_0$ for all four approaches. Estimates given in $\mu g/g$. The simulation is based on the parameters which are deduced from the FAES dataset for Na given in Table 4 (for more information on the simulations, see text below)

**Table 3:** Application of the different approaches and the respective approximation formulas to the FAES dataset [13]

Element	Approach	$\hat{C}_0$	$s_{\hat{C}_0}$	$\widehat{bias}$	$CI_l$	$CI_u$	CI width
Na$_{589.0 \, \text {nm}}$	Extrapolation	19.72	0.63	0.008	18.41	21.03	2.63
Na$_{589.0 \, \text {nm}}$	Interpolation	21.34	0.92	0.008	19.45	23.23	3.79
Na$_{589.0 \, \text {nm}}$	Reversed axis	19.53	0.63	$-$0.181	18.22	20.84	2.61
Na$_{589.0 \, \text {nm}}$	Normalization	21.02	0.34	$-$0.011	20.32	21.73	1.41
K$_{766.5 \, \text {nm}}$	Extrapolation	24.02	0.46	0.004	23.06	24.98	1.91
K$_{766.5 \, \text {nm}}$	Interpolation	24.19	0.63	0.004	22.88	25.50	2.61
K$_{766.5 \, \text {nm}}$	Reversed axis	23.92	0.46	$-$0.089	22.97	24.88	1.91
K$_{766.5 \, \text {nm}}$	Normalization	24.16	0.48	$-$0.018	23.16	25.16	2.01
K$_{769.9 \, \text {nm}}$	Extrapolation	22.65	0.63	0.008	21.34	23.95	2.60
K$_{769.9 \, \text {nm}}$	Interpolation	23.30	0.87	0.007	21.50	25.11	3.61
K$_{769.9 \, \text {nm}}$	Reversed axis	22.47	0.63	$-$0.169	21.17	23.77	2.59
K$_{769.9 \, \text {nm}}$	Normalization	23.19	0.52	$-$0.021	22.12	24.26	2.14

Values given in $\mu g/g$

### Real-world example and simulations

This subsection provides in addition to the analysis of a real data set the results of some simulations based on this data set. Especially, the simulations serve two different purposes, firstly to validate the approximation formulas by showing that the approximations and the simulations yield reasonably close results and secondly to enable the comparison of the different standard addition approaches.

The real-world example is taken from the paper of Gonçalves et al. [13]. They compared the extrapolation approach and reverse regression for Na and K determination in biodiesel based on measurements generated by applying FAES. The results of the analysis of these FAES data with respect to all four approaches can be found in Table 3 which shows estimates for $C_0, \sigma ^2_{\hat{C}_0}$ and the *bias* and also the lower and upper bounds of the CIs, as well as their width. These estimates are denoted $\hat{C}_0, s_{\hat{C}_0}, \widehat{bias}, CI_l, CI_u$ and CI width.

In the case of the FAES data, the assumption of homoscedastic errors seems to apply. Therefore, the derived formulas have been used to estimate bias and variance for all considered methods by replacing the true (unknown) values of the parameters of the underlying relationship by the respective estimates. The variance estimates $s^2_{\hat{C}_0}$ have further been used to calculate the confidence intervals which has been done based on the following assumptions: Since the estimator $\hat{C}_0$ can be assumed to be approximately normally distributed (see Fig. 3), we assume that $\frac{\hat{C}_0 - C_0}{s_{\hat{C}_0}} \sim t_{n-n_{p}}$, i.e., that this fraction is distributed according to Student’s *t*-distribution with $n - n_p$ degrees of freedom (*df*). $n_p$ equals the number of estimated parameters, i.e., $n_p=2$ for all approaches (but see the discussion on the normalization method). Therefore, the proper confidence interval should be given by $\hat{C}_0 \pm s_{\hat{C}_0}t_{n-n_p,1-\alpha /2}$ with $1-\alpha$ denoting the chosen confidence level of the CI and $t_{n-n_p,1-\alpha /2}$ denoting the $1-\alpha /2$ quantile of the *t*-distribution with $df = n - n_p$.

Furthermore, the FAES data are also used to deduce the parameters for the simulations whose results are shown in Table 5. These parameters are estimated by applying linear regression to the FAES data and are shown in Table 4. For these simulations, also, the spiked concentrations $\varvec{x}$ chosen by Gonçalves et al. are used, which are as follows: $n_e=n_r=5$ and thus $n=n_en_r=25$ with $(x_{r1},...,x_{r5})=(0, 11.4, 23, 34.5, 45.9)$ and $r=1,...,5$.

**Table 4:** Elementwise estimated parameters for the FAES dataset [13]

Element	$\hat{\beta }_0$	$\hat{\beta }_1$	$\hat{\sigma }$	$\hat{\beta }_0/\hat{\beta }_1$ (true $C_0$ in simulations given in $\mu g/g$)
Na$_{589.0 \, \text {nm}}$	4016.98	203.71	229.96	19.72
K$_{766.5 \, \text {nm}}$	3603.00	150.01	113.49	24.02
K$_{769.9 \, \text {nm}}$	1348.53	59.55	62.86	22.65

**Table 5:** Results of applying the approximation formulas and simulations ($10^4$ iterations) to the parameters (Table 4) deduced from the FAES dataset [13]

Element	Approach	$\overline{\hat{C}}_{0 sim}$	$bias_{appr}$	$\sigma _{\hat{C}_0 appr}$	$bias_{sim}$	$\sigma _{\hat{C}_0 sim}$	$CI_{cov sim}$	$\overline{CI}_{sim}$
Na$_{589.0 \, \text {nm}}$	Extrapolation	19.73	0.008	0.63	0.006	0.63	0.952	2.60
Na$_{589.0 \, \text {nm}}$	Interpolation	19.72	0.007	0.90	0.003	0.90	0.959	3.69
Na$_{589.0 \, \text {nm}}$	Reversed axis	19.54	$-$0.181	0.63	$-$0.176	0.63	0.940	2.59
Na$_{589.0 \, \text {nm}}$	Normalization	19.65	$-$0.068	0.81	$-$0.066	0.81	0.984	6.16
K$_{766.5 \, \text {nm}}$	Extrapolation	24.03	0.004	0.46	0.013	0.46	0.949	1.90
K$_{766.5 \, \text {nm}}$	Interpolation	24.02	0.004	0.63	0.003	0.64	0.955	2.58
K$_{766.5 \, \text {nm}}$	Reversed axis	23.93	$-$0.090	0.46	$-$0.083	0.46	0.946	1.89
K$_{766.5 \, \text {nm}}$	Normalization	23.99	$-$0.026	0.59	$-$0.028	0.58	0.985	4.42
K$_{769.9 \, \text {nm}}$	Extrapolation	22.65	0.008	0.63	0.008	0.63	0.948	2.57
K$_{769.9 \, \text {nm}}$	Interpolation	22.65	0.007	0.87	0.001	0.87	0.956	3.55
K$_{769.9 \, \text {nm}}$	Reversed axis	22.49	$-$0.169	0.63	$-$0.153	0.63	0.942	2.57
K$_{769.9 \, \text {nm}}$	Normalization	22.60	$-$0.053	0.80	$-$0.050	0.80	0.986	6.06

Values given in $\mu g/g$

All simulations (as already stated, many more than those whose results are shown in this work have been performed) are based on the assumption, that the true parameters (*y*-intercept $\beta _0$, slope $\beta _1$ and measurement error $\sigma$), and therefore, the true relationship and especially $C_0$ is known. With respect to the simulation results in Table 5, this means that $\beta _0$, $\beta _1$ and $\sigma$ have been chosen to be $\hat{\beta }_0$, $\hat{\beta }_1$ and $\hat{\sigma }$ from Table 4. Therefore, $bias_{appr}$ and $\sigma _{\hat{C}_0 appr}$ shown in Table 5 approximating the true values of *bias* and $\sigma ^2_{\hat{C}_0}$ can be calculated just by plugging in the known parameters together with the chosen spiked concentrations given by $\varvec{x}$ into the respective formulas in Tables 1 and 2.

To get robust simulation results, each of these results is based on the outcomes of a large number *K* of iterations which has been chosen to be $10^4$ in this case. In each such iteration $k, k=1,...,K,$ the spiked concentrations $\varvec{x}$ together with the parameters are used to generate $n=n_rn_e$ new synthetic random measurements by applying the relationship $Y_{ri}=\beta _0+\beta _1x_{ri}+\varepsilon$ with $\varepsilon \sim N(0,\sigma ), \ r=1,...,n_r, \ i=1,...,n_e$. Each of these newly generated sets of *n* measurements $\varvec{y_k}=(y_{11},...,y_{n_rn_e})$ is subsequently analyzed using the four standard addition approaches to estimate $C_0$ and also plugging in the proper estimates into the formulas given in Table 2 to calculate CIs, i.e., in each iteration *k*, there is a new estimate $\hat{C}_{0 k}$ and also a new CI, $CI_k$, for $C_0$ calculated. Therefore, the quantities found in Table 5 are calculated as follows:

$$\begin{aligned} \overline{\hat{C}}_{0 sim}&=\frac{1}{K}\sum ^{K}_{k=1}\hat{C}_{0 k}, \quad bias_{sim}=C_0-\overline{\hat{C}}_{0 sim}, \quad \sigma ^2_{\hat{C}_0 sim}\\&=\frac{1}{K-1}\sum ^{K}_{k=1}(\hat{C}_{0 k}-\overline{\hat{C}}_{0 sim})^2, \end{aligned}$$

$CI_{cov sim}$ is the fraction of all $CI_k$ covering $C_0$, and $\overline{CI}_{sim}$ is the mean width of all $CI_k$.

## Discussion

### Analysis of the FAES data

The results with respect to the analysis of the FAES data shown in Table 3 vary considerably with respect to the estimated concentration $\hat{C}_0$ (e.g., it can be shown that $\hat{C}_0^e - \hat{C}_0^r \approx \widehat{bias}_e - \widehat{bias}_r$). Also, the results with respect to the performance criteria, i.e., bias and variance of $\hat{C}_0$, show significant differences between the approaches. The estimated variances $s^2_{\hat{C}_0}$ and therefore the width of the respective CIs behave as anticipated except for the normalization approach, but that $s^2_{\hat{C}_0^n}$ is close to or even smaller than $s^2_{\hat{C}_0^e}$ is only due to chance. As already stated, assessment of an approach is not possible based on just one data set, i.e., to get a clear picture, the approximation formulas or simulations are needed.

**Table 6:** Quick overview of the results of the comparison of the four approaches in terms of bias and precision for the case of normally distributed homoscedastic errors

Approach	Extrapolation	Interpolation	Reversed axis	Normalization
Bias				
Precision				

### Validation of the approximation formulas and comparison of the approaches

Numerous simulations have been applied to validate the approximation formulas which also allows in parallel to investigate the performance of the approaches. For validation of the formulas, the values calculated by using these formulas have to be compared to the respective simulation results, which should be very close to the true values. Hence, for the values given in Table 5, the values for $bias_{appr}$ and $\sigma _{\hat{C}_0 appr}$ have to be compared to the values for $bias_{sim}$ and $\sigma _{\hat{C}_0 sim}$. Since the approximations are very close to the simulation results for bias and variance of the different approaches, all four approaches can be judged either based on the approximations or the simulations. Therefore, the conclusions with respect to all considered parameter settings (not just the settings shown in Table 4) are the same whether they are based on the approximation formulas or the simulations.

The following observations were made with regard to bias: First of all, the extra- and interpolation approach show a positive bias (systematic overestimation of $C_0$), and the reversed-axis and the normalization approach show a negative bias (systematic underestimation of $C_0$). The extrapolation approach together with the interpolation approach shows, in absolute terms, the smallest bias, the reversed-axis approach shows the largest, and the normalization approach is somewhere in between. The approximation formulas are consistent with the simulation results (see again Table 5 for some of these results) for the reversed-axis and the normalization approach and the respective simulation results fluctuate in a range that is in line with the order of magnitude indicated by the approximation formulas for the extrapolation and the interpolation approach. We think that the fluctuations in the simulations for the latter two cases are due to the relatively small bias compared to the variance of the estimator.

The following observations were made with regard to variance: The extrapolation approach has the smallest variance which is equal to that of the reversed-axis approach, whereas the interpolation approach in contradiction to its intention shows the largest variance. This effect with respect to $\sigma ^2_{\hat{C}_0^i}$ is due to the additional variance introduced by $y_{1}$, i.e., this approach has an adverse effect due to an additional source of variability (see Fig. 2b and also the description of this approach in the “Methods” section). The normalization approach shows a variance which is also significantly larger than that of the extrapolation approach and can be found somewhere in between that of the extra- and the interpolation approach. The approximation formulas work well for all approaches, i.e., they are pretty close to the results gained by simulations.

The following observations were made with regard to the confidence intervals: In the case of Table 5, the respective results given by simulations are contained in the columns $CI_{cov sim}$ (coverage probability) and $\overline{CI}_{sim}$ (average width). It is of interest whether these intervals cover the true concentration with a probability determined beforehand by choosing the desired level of confidence. Another important feature is the width of the CIs since one, of course, wants them to be as narrow as possible. Since the CI width is a direct consequence of $s^2_{\hat{C}_0}$, the CIs are narrowest for the extrapolation- and the reversed-axis approach. Different confidence levels have been investigated by using simulations. In Table 5, results are shown for a chosen confidence level of 95%. The CIs covered the true concentration for all approaches, except for the normalization approach, approximately with the desired probability. Of course, the larger the bias, the less accurate are the respective CIs. Especially in the case of the reversed-axis approach, this might cause a (slightly) decreased probability of covering the true value $C_0$ due to the increased (negative) bias of this approach (correcting for the bias by incorporating $\widehat{bias}$ in the CI calculation might be of interest). In the normalization case, the coverage probability is higher than the desired confidence level due to the already stated problem of overestimating the variance of $\hat{C}_0$, which has the consequence of overly wide CIs (see discussion above).

Therefore, taking all of the above together, the most recommendable approach with respect to bias and variance is still the common extrapolation approach. The results with respect to the comparison of the different approaches are briefly summarized in Table 6.

## Summary

Simulations yield that, in comparison to the common extrapolation approach, all the other approaches show a decreased performance with respect to bias and/or variance of the estimator for the unknown concentration when the measurement errors are normally distributed and homoscedastic. Especially, the interpolation approach does not seem to be recommendable as it does not increase the bias of the respective estimator, but in contradiction to the intention of reducing its variance, it significantly increases its variability compared to the estimators resulting from the extrapolation and the reversed-axis approach. For the latter two approaches, the variance of the estimators is very similar. Therefore, the reversed-axis approach works well with regard to the intended simplification of the determination of the variance of $\hat{C}_0$. The disadvantage of this approach is that it has the greatest bias of all approaches in absolute terms. Since this bias is of negative sign, the reversed-axis approach underestimates the true concentration on average more than the extrapolation approach, whose bias is positive, overestimates it. In addition, the increased bias also has a slight impact on the CI calculated with respect to reverse regression, i.e., the achieved confidence level is slightly lower than the chosen one. The normalization approach has an exceptional position since performance with respect to bias and variance is weaker than that of the extrapolation approach, but it can handle outliers better. Therefore, a trade-off must be found between greater robustness to outliers (in cases where the unspiked measurements are not affected and the outliers cannot be removed before analysis) and the price of greater bias and variance. An additional problem when applying the normalization approach is the estimation of the variance of the estimator. Depending on the method applied, this can lead to CIs which are wastefully wide, i.e., reaching a confidence level significantly higher than chosen beforehand.

The derived approximation formulas, which are an additional outcome of this investigation, proved to be valid, i.e., they are in very good agreement with the simulation results. This allows the calculation of CIs also in the case of inverse regression and the normalization approach and can replace the use of simulations when investigating the performance of the different standard addition approaches.

## Supplementary Information

Below is the link to the electronic supplementary material.

Supplementary file 1 (pdf 597 KB)
