# Supplementary material for: Evaluation of four different standard addition approaches with respect to trueness and precision
Source: Anal Bioanal Chem. 2025 Jan 10;417(6):1187–97. doi: 10.1007/s00216-024-05725-8 (PMC11802646; doi:10.1007/s00216-024-05725-8)
Supplement: Supplementary file 1 — (pdf 597 KB) [file 216_2024_5725_MOESM1_ESM.pdf]

Supplement for:

Evaluation of four different standard addition approaches with respect to trueness and precision

by

Gerhard Gössler, Vera Hofer, and Walter Goessler

Content

Bias Extrapolation Approach 2
Bias Interpolation Approach 4
Bias Normalization Approach 7
Variance Interpolation Method 9
Variance Normalization Approach 13

Bias Extrapolation Approach

$$Y = \beta_0 + \beta_1 x + \varepsilon$$

$$C_0 = \beta_0/\beta_1$$

$$\hat{\beta}_1 = S_{xy}/S_{xx}$$

$$\hat{\beta}_0 = \bar{Y} - \hat{\beta}_1 \bar{x}$$

$$\hat{C}_0 = \hat{\beta}_0/\hat{\beta}_1$$

$$E(\hat{C}_0) = E(\hat{\beta}_0)E\left(\frac{1}{\hat{\beta}_1}\right) + cov\left(\hat{\beta}_0, \frac{1}{\hat{\beta}_1}\right) =$$

$$= E(\hat{\beta}_0)E\left(\frac{1}{\hat{\beta}_1}\right) + cov\left(\bar{Y} - \hat{\beta}_1\bar{x}, \frac{1}{\hat{\beta}_1}\right) = E(\hat{\beta}_0)E\left(\frac{1}{\hat{\beta}_1}\right) + cov\left(\bar{Y}, \frac{1}{\hat{\beta}_1}\right) - \bar{x}cov\left(\hat{\beta}_1, \frac{1}{\hat{\beta}_1}\right)$$

$$E\left(\frac{1}{\hat{\beta}_1}\right) = ? \quad , \quad cov\left(\hat{\beta}_0, \frac{1}{\hat{\beta}_1}\right) = ?$$

$$cov\left(\bar{Y}, \frac{1}{\hat{\beta}_1}\right) = ? \quad , \quad cov\left(\hat{\beta}_1, \frac{1}{\hat{\beta}_1}\right) = ?$$

$\boldsymbol{E\left(\frac{1}{\hat{\beta}_1}\right)}$

$$1 = E(1) = E(\hat{\beta}_1)E(1/\hat{\beta}_1) + cov(\hat{\beta}_1, 1/\hat{\beta}_1)$$

$$E(1/\hat{\beta}_1) = (1 - cov(\hat{\beta}_1, 1/\hat{\beta}_1))/\, E(\hat{\beta}_1)$$

$$1 = F = \hat{\beta}_1 \cdot 1/\hat{\beta}_1 = AB$$

$$0 = var(F) = B^2\sigma_A^2 + A^2\sigma_B^2 + 2ABcov(A, B)$$

$$\boldsymbol{cov(A, B)} = -\frac{B^2\sigma_A^2 + A^2\sigma_B^2}{2AB}$$

$$A = S_{xy}/S_{xx}, \quad B = S_{xx}/S_{xy}$$

$$A = \frac{S_{xy}}{S_{xx}} = \frac{\sum(x_i - \bar{x})y_i}{\sum(x_i - \bar{x})^2}$$

$$\frac{\partial A}{\partial y_i} = \frac{x_i - \bar{x}}{\sum(x_i - \bar{x})^2}$$

$$B = \frac{S_{xx}}{S_{xy}} = \frac{\sum(x_i - \bar{x})^2}{\sum(x_i - \bar{x})y_i}$$

$$\frac{\partial B}{\partial y_i} = -\frac{(x_i - \bar{x})\sum(x_i - \bar{x})^2}{(\sum(x_i - \bar{x})y_i)^2}$$

$$\left(\frac{\partial B}{\partial y_i}\right)^2 = \frac{(x_i - \bar{x})^2 S_{xx}^2}{S_{xy}^4}$$

$$\boldsymbol{\sigma_B^2} \approx \sum_{i=1}^{n}\left(\frac{\partial B}{\partial y_i}\right)^2 \sigma^2 = \frac{\sum_{i=1}^{n}(x_i - \bar{x})^2 S_{xx}^2}{S_{xy}^4} = \boldsymbol{\frac{S_{xx}^3}{S_{xy}^4}\sigma^2}$$

$$cov(A,B) = -\frac{B^2\sigma_A^2 + A^2\sigma_B^2}{2AB}$$

$$B^2\sigma_A^2 + A^2\sigma_B^2 = \frac{S_{xx}^2}{S_{xy}^2}\frac{\sigma^2}{S_{xx}} + \frac{S_{xy}^2}{S_{xx}^2}\frac{S_{xx}^3}{S_{xy}^4}\sigma^2 = \sigma^2\left(\frac{S_{xx}}{S_{xy}^2} + \frac{S_{xx}}{S_{xy}^2}\right) = 2\sigma^2\frac{S_{xx}}{S_{xy}^2}$$

$$\boldsymbol{cov\left(\hat{\beta}_1, \frac{1}{\hat{\beta}_1}\right) = cov(A,B)} = |AB = 1| = \boldsymbol{-\sigma^2\frac{S_{xx}}{S_{xy}^2}}$$

$$1 = E(1) = E(\hat{\beta}_1)E(1/\hat{\beta}_1) + cov(\hat{\beta}_1, 1/\hat{\beta}_1)$$

$$E(1/\hat{\beta}_1) = (1 - cov(\hat{\beta}_1, 1/\hat{\beta}_1))/\ E(\hat{\beta}_1)$$

$$E(\hat{\beta}_1) = b$$

$$\boldsymbol{E(1/\hat{\beta}_1) = \left(1 + \sigma^2\frac{S_{xx}}{S_{xy}^2}\right)/\beta_1}$$

$$\boldsymbol{cov\left(\hat{\beta}_0, \frac{1}{\hat{\beta}_1}\right)}$$

$$cov\left(\hat{\beta}_0, \frac{1}{\hat{\beta}_1}\right) = cov\left(\bar{Y} - \hat{\beta}_1\bar{x}, \frac{1}{\hat{\beta}_1}\right) = cov\left(\bar{Y}, \frac{1}{\hat{\beta}_1}\right) - \bar{x}cov\left(\hat{\beta}_1, \frac{1}{\hat{\beta}_1}\right)$$

$$cov\left(\bar{Y},\frac{1}{\hat{\beta}_1}\right)=cov\left(\frac{1}{n}\sum y_i,\frac{1}{\hat{\beta}_1}\right)=\frac{1}{n}\sum cov\left(y_i,\frac{1}{\hat{\beta}_1}\right)=\frac{1}{n}\sum cov\left(y_i,\frac{S_{xx}}{S_{xy}}\right)=$$

$$\frac{S_{xx}}{n}\sum cov\left(y_i,\frac{1}{S_{xy}}\right)=\frac{S_{xx}}{n}\sum cov\left(y_i,\frac{1}{\sum(x_i-\bar{x})\,y_i}\right)\approx 0$$

$cov\left(\bar{Y},\frac{1}{\hat{\beta}_1}\right)\approx 0$ follows from $(x_1-\bar{x})=-(x_n-\bar{x}),\ldots,\left(x_{n/2}-\bar{x}\right)=-\left(x_{n/2+1}-\bar{x}\right)$: due to this property the $cov\left(y_i,\frac{1}{\sum(x_i-\bar{x})y_i}\right)$ cancel each other out.

$$\Downarrow$$

$$cov\left(\hat{\beta}_0,\frac{1}{\hat{\beta}_1}\right)\approx -\bar{x}cov\left(\hat{\beta}_1,\frac{1}{\hat{\beta}_1}\right)$$

Bias $\widehat{\boldsymbol{C}}_{\boldsymbol{0}}$

$$E(\hat{C}_0)=E(\hat{\beta}_0)E\left(\frac{1}{\hat{\beta}_1}\right)+cov\left(\hat{\beta}_0,\frac{1}{\hat{\beta}_1}\right)\approx E(\hat{\beta}_0)E\left(\frac{1}{\hat{\beta}_1}\right)-\bar{x}cov\left(\hat{\beta}_1,\frac{1}{\hat{\beta}_1}\right)$$

$$\Downarrow$$

$$\boldsymbol{E}(\widehat{\boldsymbol{C}}_{\boldsymbol{0}})\approx\frac{\beta_0\left(1+\sigma^2\frac{S_{xx}}{S_{xy}^2}\right)}{\beta_1}+\sigma^2\bar{x}\frac{S_{xx}}{S_{xy}^2}=C_0+C_0\sigma^2\frac{S_{xx}}{S_{xy}^2}+\sigma^2\bar{x}\frac{S_{xx}}{S_{xy}^2}=\boldsymbol{C_0}+\boldsymbol{\sigma^2}\frac{\boldsymbol{S_{xx}}}{\boldsymbol{S_{xy}^2}}(\boldsymbol{C_0}+\bar{\boldsymbol{x}})$$

Bias Interpolation Approach

$$\hat{C}_0=\frac{(2\bar{y}_1-\hat{\beta}_0)}{\hat{\beta}_1}=\frac{\hat{\beta}_0^i}{\hat{\beta}_1}$$

$$\hat{\beta}_1=\frac{S_{xy}}{S_{xx}}\quad,\quad\hat{\beta}_0=\bar{Y}-\hat{\beta}_1\bar{x}$$

$$2\bar{Y}_1-\hat{\beta}_0=2\bar{Y}_1-\bar{Y}+\hat{\beta}_1\bar{x}$$

$$\bar{Y}_1=\frac{1}{n_r}\sum_{i=1}^{n_r}Y_{i1}\quad,\quad\bar{Y}=\frac{1}{n}\sum_{i=1}^{n_r}\sum_{j=1}^{n_e}Y_{ji}\quad,\quad n=n_rn_e$$

$$E\left(\frac{\hat{\beta}_0^i}{\hat{\beta}_1}\right) = E(\hat{\beta}_0^i)E\left(\frac{1}{\hat{\beta}_1}\right) + cov\left(\hat{\beta}_0^i, \frac{1}{\hat{\beta}_1}\right)$$

$$\boldsymbol{cov}\left(\boldsymbol{\hat{\beta}_0^i}, \frac{\mathbf{1}}{\boldsymbol{\hat{\beta}_1}}\right) = cov\left(2\bar{Y}_1 - \bar{Y} + \hat{\beta}_1\bar{x}, \frac{1}{\hat{\beta}_1}\right) = 2cov\left(\bar{Y}_1, \frac{1}{\hat{\beta}_1}\right) - cov\left(\bar{Y}, \frac{1}{\hat{\beta}_1}\right) + \bar{x}cov\left(\hat{\beta}_1, \frac{1}{\hat{\beta}_1}\right) =$$

$$\frac{2}{n_r}cov\left(Y_{11} + \cdots + Y_{n_r1}, \frac{1}{\hat{\beta}_1}\right) - cov\left(\bar{Y}, \frac{1}{\hat{\beta}_1}\right) + \bar{x}cov\left(\hat{\beta}_1, \frac{1}{\hat{\beta}_1}\right)$$

$$\frac{2}{n_r}cov\left(Y_{11} + \cdots + Y_{n_r1}, \frac{1}{\hat{\beta}_1}\right) = 2cov\left(Y_{i1}, \frac{1}{\hat{\beta}_1}\right) \quad , \quad i = 1, \dots, n_r$$

$$\boldsymbol{cov}\left(\boldsymbol{Y_{11}}, \frac{\mathbf{1}}{\boldsymbol{\hat{\beta}_1}}\right) = S_{xx}cov\left(Y_{11}, \frac{1}{\sum_{i=1}^{n_r}\sum_{j=1}^{n_e}(x_i - \bar{x})Y_{ij}}\right) = S_{xx}cov\left(Y_{11}, \frac{1}{k_{11} + k_{21}Y_{11}}\right) ,$$

$$k_{21} = (x_1 - \bar{x})$$

Taylor-Series

$$\left(\frac{1}{k_{11} + k_{21}Y_{11}}\right)' = -\frac{k_{21}}{(k_{11} + k_{21}Y_{11})^2} = t_1$$

$$\left(-\frac{k_{21}}{(k_{11} + k_{21}Y_{11})^2}\right)' = \frac{k_{21}^2}{(k_{11} + k_{21}Y_{11})^4} = t_2$$

$$\left(\frac{k_{21}^2}{(k_{11} + k_{21}Y_{11})^4}\right)' = -\frac{k_{21}^3}{(k_{11} + k_{21}Y_{11})^8} = t_3$$

$y_0 = a + bx$, $y_{110} = E(Y_{11})$, k_{110}, t_1, t_2, t_3 ... evaluated for $y_{110}, \dots, y_{n_r n_e 0}$

$$\Downarrow$$

$$\frac{1}{k_{11} + k_{21}Y_{11}} \approx \frac{1}{k_{110} + k_{21}y_{110}} + t_1(Y_{11} - y_{110}) + \frac{t_2(Y_{11} - y_{110})^2}{2} + \frac{t_3(Y_{11} - y_{110})^3}{6}$$

since t_2 and t_3 are very small

$$\Downarrow$$

$$\frac{1}{k_{11} + k_{21}y_{11}} \approx \frac{1}{k_{110} + k_{21}y_{110}} + t_1(Y_{11} - y_{110}) = \frac{1}{cov(x, y_0)} + t_1(Y_{11} - y_{110})$$

$$\Downarrow$$

$$cov\left(Y_{11},\frac{1}{k_{11}+k_{21}Y_{11}}\right)\approx cov\left(y_{11},\frac{1}{cov(x,y_0)}+t_1(y_{11}-y_{110})\right)=$$

$$cov(y_{11},t_1y_{11})=t_1cov(y_{11},y_{11})=t_1\sigma^2\quad,\quad t_1=\frac{\bar{x}-x_1}{S_{xy}^2}$$

$$\Downarrow$$

$$\boldsymbol{cov\left(Y_{11},\frac{1}{\hat{\beta}_1}\right)}=S_{xx}cov\left(Y_{11},\frac{1}{\sum(x_i-\bar{x})\,y_{ji}}\right)=S_{xx}cov\left(Y_{11},\frac{1}{k_{11}+k_{21}y_{11}}\right)$$

$$\approx \boldsymbol{\sigma^2S_{xx}(\bar{x}-x_1)/S_{xy}^2}$$

$$cov\left(\hat{\beta}_0^i,\frac{1}{\hat{\beta}_1}\right)=\frac{2}{n_r}cov\left(Y_{11}+\cdots+Y_{n_r1},\frac{1}{\hat{\beta}_1}\right)-cov\left(\bar{Y},\frac{1}{\hat{\beta}_1}\right)+\bar{x}cov\left(\hat{\beta}_1,\frac{1}{\hat{\beta}_1}\right)$$

$cov\left(\bar{Y},\frac{1}{\hat{\beta}_1}\right)\approx 0$ (see derivation of the bias for the extrapolation approach),

$cov\left(\hat{\beta}_1,\frac{1}{\hat{\beta}_1}\right)\approx -\sigma^2\frac{S_{xx}}{S_{xy}^2}$ (see derivation of the bias for the extrapolation approach)

$$E(\hat{\beta}_0^i)=\beta_0\quad,\quad E\left(\frac{1}{\hat{\beta}_1}\right)\approx\frac{\left(1+\sigma^2\frac{S_{xx}}{S_{xy}^2}\right)}{\beta_1}\quad,\qquad\frac{\beta_0}{\beta_1}=C_0$$

$$\Downarrow$$

$$\boldsymbol{cov\left(\hat{\beta}_0^i,\frac{1}{\hat{\beta}_1}\right)}\approx\frac{2\sigma^2S_{xx}(\bar{x}-x_1)}{S_{xy}^2}-\sigma^2\bar{x}\frac{S_{xx}}{S_{xy}^2}=\sigma^2\frac{S_{xx}}{S_{xy}^2}(2(\bar{x}-x_1)-\bar{x})=\sigma^2\frac{S_{xx}}{S_{xy}^2}(\bar{x}-x_1)=$$

$$\boldsymbol{\sigma^2\bar{x}\frac{S_{xx}}{S_{xy}^2}}\ \text{since}\ x_1=0$$

$$\Downarrow$$

$$\boldsymbol{E\left(\frac{\hat{\beta}_0^i}{\hat{\beta}_1}\right)}=E(\hat{\beta}_0^i)E\left(\frac{1}{\hat{\beta}_1}\right)+cov\left(\hat{\beta}_0^i,\frac{1}{\hat{\beta}_1}\right)\approx E(\hat{\beta}_0^i)E\left(\frac{1}{\hat{\beta}_1}\right)+\sigma^2\bar{x}\frac{S_{xx}}{S_{xy}^2}=$$

$$\beta_0\frac{\left(1+\sigma^2\frac{S_{xx}}{S_{xy}^2}\right)}{\beta_1}+\sigma^2\bar{x}\frac{S_{xx}}{S_{xy}^2}=C_0+C_0\sigma^2\frac{S_{xx}}{S_{xy}^2}+\sigma^2\bar{x}\frac{S_{xx}}{S_{xy}^2}=\boldsymbol{C_0+\sigma^2\frac{S_{xx}}{S_{xy}^2}(C_0+\bar{x})}$$

Bias Normalization Approach

Calculation of the Bias of the estimator for C_0 ($\hat{C}_0 = 1/\hat{\beta}$).

The model

All observed values

$$Y = \beta_0 + \beta_1 x + \varepsilon$$

of each series are divided by the first observation of each series, i.e., divided by

$$Y_1 = \beta_0 + \varepsilon_1.$$

Therefore, the normalized observations $\tilde{y}$ are given by

$$y^n = \frac{y}{y_1} = \frac{\beta_0}{\beta_0 + \varepsilon_1} + \frac{x\beta_1}{\beta_0 + \varepsilon_1} + \frac{\varepsilon}{\beta_0 + \varepsilon_1} = \beta_0^n + \beta_1^n x + \varepsilon^n$$

and we have that $y_1^n = \frac{y_1}{y_1} = 1$ for each series.

Since $y_1^n = 1$ for each series, the regression line has to be forced through $(0,1)$, i.e.,

$$y^n = 1 + \beta x + \varepsilon^r$$

estimation of the intercept is not necessary and therefore only an estimator for the slope is needed, i.e., the following sum of squared errors has to be minimized with respect to β (n is the number of observations):

$$SSE = \sum \left(y_i^n - \overline{y}^n\right)^2 = \sum \left(y_i^n - 1 - \hat{\beta} x_i\right)^2$$

$$\frac{\partial}{\hat{\beta}} \sum \left(y_i^n - 1 - \hat{\beta} x_i\right)^2 = -2 \sum \left(y_i^n - 1 - \hat{\beta} x_i\right) x_i = -2 \sum \left((y_i^n - 1) x_i - \hat{\beta} x_i^2\right) =$$

$$-2 \sum (y_i^n - 1) x_i + 2\hat{\beta} \sum x_i^2$$

$$\Downarrow$$

$$\hat{\beta} = \frac{\sum (y_i^n - 1) x_i}{\sum x_i^2} = \frac{\sum y_i^n x_i - n\bar{x}}{\sum x_i^2} = \frac{1}{\sum x_i^2} \sum y_i^n x_i - \frac{n\bar{x}}{\sum x_i^2}$$

Now, let's consider that n_r different series have been measured and that each series i consists of n_e single observations $y_{ij}, i = 1, \dots, n_r, j = 1, \dots, n_e$, i.e., $n = n_e n_r$, then

$$\sum_{l=1}^{n} y_l^n x_l = \frac{1}{y_{11}} \sum_{j=1}^{n_e} y_{1j} x_{1j} + \dots + \frac{1}{y_{1n_r}} \sum_{j=1}^{n_e} y_{n_r j} x_{1j}$$

The bias of $\hat{C}_0 = 1/\hat{\beta}$ is given by

$$Bias(\hat{C}_0) = C_0 - E(\hat{C}_0) = \frac{\beta_0}{\beta_1} - E\left(\frac{1}{\hat{\beta}}\right)$$

Approximation of the bias based on the second-order Taylor polynomial of $\frac{1}{\hat{\beta}}$

$$\frac{1}{\hat{\beta}} = \frac{\sum x_i^2}{\sum y_i^n x_i - n\bar{x}} = \frac{\sum x_i^2}{1/y_{11} \sum_{i=2}^{n_e} y_{1i} x_i + x_1 + K - n\bar{x}}$$

$$1/y_{11} \sum_{i>1} y_{i1} x_i + x_1 + K - n\bar{x} = \hat{\beta} \sum x_i^2$$

$$\frac{\partial}{\partial y_{11}} \left(\frac{1}{1/y_{11} \sum_{i>1} y_{1i} x_i + x_1 + K - n\bar{x}} \right) = \frac{1/y_{11}^2 \sum_{i>1} y_{1i} x_i}{(1/y_{11} \sum_{i>1} y_{1i} x_i + x_1 + K - n\bar{x})^2}$$

$$\Rightarrow \frac{\partial}{\partial y_{11}} \left(\frac{1}{\hat{\beta}} \right) = \frac{1/y_{11}^2 \sum_{i>1} y_{1i} x_i}{\hat{\beta}^2 \sum x_i^2}$$

$$\frac{\partial}{\partial y_{11}^2} \left(\frac{1}{1/y_{11} \sum_{i>1} y_{i1} x_i + x_1 + K - n\bar{x}} \right) =$$

$$= \frac{-2 \frac{\sum_{i>1} y_{1i} x_i \, (1/y_{11} \sum_{i>1} y_{i1} x_i + x_1 + K - n\bar{x})^2}{y_{11}^3} + 2(1/y_{11} \sum_{i>1} y_{1i} x_i + x_1 + K - n\bar{x})(1/y_{11}^2 \sum_{i>1} y_{1i} x_i)^2}{(1/y_{11} \sum_{i>1} y_{1i} x_i + x_1 + K - n\bar{x})^4}$$

$$= \frac{-2 \frac{\sum_{i>1} y_{1i} x_i \left(\hat{\beta} \sum x_i^2\right)^2}{y_{11}^3} + 2\hat{\beta} \sum x_i^2 \, (1/y_{11}^2 \sum_{i>1} y_{1i} x_i)^2}{\left(\hat{\beta} \sum x_i^2\right)^4}$$

$$\frac{\partial}{\partial y_{11}^2} \left(\frac{1}{\hat{\beta}} \right) = \frac{-2 \frac{\sum_{i>1} y_{i1} x_i \left(\hat{\beta} \sum x_i^2\right)^2}{y_{11}^3} + 2\hat{\beta} \sum x_i^2 \, (1/y_{11}^2 \sum_{i>1} y_{i1} x_i)^2}{\hat{\beta} \left(\hat{\beta} \sum x_i^2\right)^3}$$

$$= \frac{-2 \frac{\hat{\beta} \sum x_i^2 \sum_{i>1} y_{i1} x_i}{y_{11}^3} + 2(1/y_{11}^2 \sum_{i>1} y_{i1} x_i)^2}{\hat{\beta} \left(\hat{\beta} \sum x_i^2\right)^2}$$

$$\frac{\partial}{\partial y_{1k}} \left(\frac{1}{1/y_{11} \sum_{i>1} y_{1i} x_i + x_1 + K - n\bar{x}} \right) = -\frac{x_k / y_{11}}{\left(\hat{\beta} \sum x_i^2\right)^2}$$

$$\frac{\partial}{\partial y_{k1}^2} \left(\frac{1}{1/y_{11} \sum_{i>1} y_{i1} x_i + x_1 + K - n\bar{x}} \right) = \frac{2(x_k/y_{11})^2 \hat{\beta} \sum x_i^2}{\left(\hat{\beta} \sum x_i^2\right)^4} = \frac{2(x_k/y_{11})^2}{\left(\hat{\beta} \sum x_i^2\right)^3}$$

$$\frac{\partial}{\partial y_{k1}^2} \left(\frac{1}{\hat{\beta}} \right) = \frac{2(x_k/y_{11})^2}{\hat{\beta} \left(\hat{\beta} \sum x_i^2\right)^2}$$

$$\boldsymbol{Bias}(\widehat{\boldsymbol{C}}_0) =$$

$$= \sigma^2 \left(n_r \frac{-\frac{\beta \sum x_i^2 \sum_{i>1} y_{1i0} x_i}{y_{110}^3} + (1/y_{110}^2 \sum_{i>1} y_{1i0} x_i)^2}{\beta\left(\beta \sum x_i^2\right)^2} + n_r \sum_{k=2}^{n_e} \frac{(x_k/y_{110})^2}{\beta\left(\beta \sum x_i^2\right)^2} \right)$$

$$= \frac{n_r \sigma^2}{\beta\left(\beta \sum x_i^2\right)^2} \left(-\frac{\beta \sum x_i^2 \sum_{i>1} y_{1i0} x_i}{y_{110}^3} + \left(1/y_{110}^2 \sum_{i>1} y_{1i0} x_i\right)^2 + \sum_{k=2}^{n_e} (x_k/y_{110})^2 \right)$$

$$= \frac{n_r \sigma^2}{\beta^3\left(\sum x_i^2\right)^2} \left(-\frac{\beta \sum x_i^2 \sum_{i>1} y_{1i0} x_i}{y_{110}^3} + \left(1/y_{110}^2 \sum_{i>1} y_{1i0} x_i\right)^2 + 1/y_{110}^2 \sum_{k=2}^{n_e} x_k^2 \right)$$

$$= \left| \beta = \frac{\beta_1}{\beta_0}, y_{110} = \beta_0 \right| =$$

$$= \frac{n_r \sigma^2 \beta_0^3}{\beta_1^3\left(\sum x_i^2\right)^2} \left(-\frac{\beta_1 \sum x_i^2 \sum_{i>1} y_{1i0} x_i}{\beta_0^4} + \left(\frac{\sum_{i>1} y_{1i0} x_i}{\beta_0^2}\right)^2 + 1/\beta_0^2 \sum_{k=2}^{n_e} x_k^2 \right)$$

$$= \frac{n_r \sigma^2 \beta_0}{\beta_1^3\left(\sum x_i^2\right)^2} \left(-\frac{\beta_1 \sum x_i^2 \sum_{i>1} y_{1i0} x_i}{\beta_0^2} + \frac{(\sum_{i>1} y_{1i0} x_i)^2}{\beta_0^2} + \sum_{k=2}^{n_e} x_k^2 \right)$$

Variance Interpolation Method

As anticipated, simulations show, that the variability $\sigma^2_{\hat{C}_0^i}$ of $\hat{C}_0^i$, the estimator of the unknown concentration C_0, is significantly larger than that of the common extrapolation approach $\sigma^2_{\hat{C}_0^e}$. This is due to the additional variability introduced by $2\bar{y}_1$. This is also shown by the following mathematical treatment of the interpolation estimator

$$\hat{C}_0^i = \frac{(2\bar{y}_1 - \hat{\beta}_0)}{\hat{\beta}_1} = \frac{\hat{\beta}_0^i}{\hat{\beta}_1}$$

$$\hat{\beta}_x = \hat{\beta}_x^e, x = 0{,}1$$

$$\bar{y}_1 = \text{mean of all unspiked measurements}$$

Since

$$E\left(\hat{\beta}_0^i\right) = E\left(2\bar{y}_1 - \hat{\beta}_0\right) = 2E(\bar{y}_1) - E(\hat{\beta}_0) = 2\beta_0 - \beta_0 = \beta_0$$

the following approximation for $\sigma^2_{\hat{C}_0^i}$ should be valid:

$$\sigma^2_{\hat{C}_0^i} \approx \left(\left(\frac{\sigma_{\hat{\beta}_0^i}}{\beta_0}\right)^2 + \left(\frac{\sigma_{\hat{\beta}_1}}{\beta_1}\right)^2 - 2\rho_{\hat{\beta}_0^i\hat{\beta}_1} \left(\frac{\sigma_{\hat{\beta}_0^i}}{\beta_0}\right)\left(\frac{\sigma_{\hat{\beta}_1}}{\beta_1}\right) \right) C_0^2$$

$$= \left|\rho_{\hat{\beta}_0^i\hat{\beta}_1} = \rho_{\hat{\beta}_0\hat{\beta}_1}\right|^* = \left(\left(\frac{\sigma_{\hat{\beta}_0^i}}{\beta_0}\right)^2 + \left(\frac{\sigma_{\hat{\beta}_1}}{\beta_1}\right)^2 - 2\rho_{\hat{\beta}_0\hat{\beta}_1} \left(\frac{\sigma_{\hat{\beta}_0}}{\beta_0}\right)\left(\frac{\sigma_{\hat{\beta}_1}}{\beta_1}\right) \right) C_0^2 =$$

$$\left(\left(\frac{\sigma_{\hat{\beta}_0^i}}{\beta_0}\right)^2 + \left(\frac{\sigma_{\hat{\beta}_1}}{\beta_1}\right)^2 - \frac{2cov\left(\hat{\beta}_0, \hat{\beta}_1\right)}{\beta_0\beta_1} \right) C_0^2 \ with\ \sigma^2_{\hat{\beta}_0^i} = var\left(\hat{\beta}_0\right) + 4\sigma^2 \left(\frac{1}{n_r} - \frac{1}{n}\left(1 + \frac{\bar{x}^2}{s_x^2}\right)\right)^*$$

* derived below

The formula above differs from the corresponding formula for the extrapolation approach with respect to $\sigma^2_{\hat{\beta}_0^i}$ and $cov(\hat{\beta}_0^i, \hat{\beta}_1)$:

$$\boldsymbol{\sigma^2_{\hat{\beta}_0^i}} = var\left(\hat{\beta}_0^i\right) = var\left(2\bar{y}_1 - \hat{\beta}_0\right) = 4var(\bar{y}_1) + var\left(\hat{\beta}_0\right) - 4cov(\bar{y}_1, \hat{\beta}_0)$$

$$\boldsymbol{cov\left(\hat{\beta}_0^i, \hat{\beta}_1\right)} = cov\left(2\bar{y}_1 - \hat{\beta}_0, \hat{\beta}_1\right) = 2cov\left(\bar{y}_1, \hat{\beta}_1\right) - cov(\hat{\beta}_0, \hat{\beta}_1)$$

$$\boldsymbol{var(\bar{y}_1) = \frac{1}{n_r}\sigma^2}$$

$$\boldsymbol{cov\left(\bar{y}_1, \hat{\beta}_0\right)} = cov\left(\bar{y}_1, \bar{Y} - \hat{\beta}_1\bar{x}\right) = cov(\bar{y}_1, \bar{Y}) - \bar{x}cov\left(\bar{y}_1, \hat{\beta}_1\right)$$

$$\boldsymbol{cov(\bar{y}_1, \bar{Y})} = cov\left(\frac{1}{n_r}\sum_{k=1}^{n_r} y_{k1}, \frac{1}{n}\sum_{i=1}^{n} y_i \right) = \boldsymbol{\frac{1}{n}\sigma^2}$$

$$\boldsymbol{cov\left(\bar{y}_1, \hat{\beta}_1\right)} = cov\left(\bar{y}_1, \frac{cov(x, Y)}{s_x^2}\right) = \frac{1}{s_x^2}cov\left(\bar{y}_1, cov(x, Y)\right)$$

$$\boldsymbol{cov(x, Y)} = \frac{1}{n-1}\sum(x_i - \bar{x})(y_i - E(y)) = \frac{1}{n-1}\sum(x_i - \bar{x})(y_i - \beta_0 - \beta_1\bar{x})$$

$$= \frac{1}{n-1}\sum(x_i - \bar{x})(\beta_0 + \beta_1 x_i + \varepsilon_i - \beta_0 - \beta_1\bar{x})$$

$$= \frac{1}{n-1}\sum(x_i - \bar{x})(\beta_1(x_i - \bar{x}) + \varepsilon_i)$$

$$= \frac{1}{n-1}\sum \beta_1(x_i - \bar{x})^2 + (x_i - \bar{x})\varepsilon_i$$

$$= \boldsymbol{\beta_1 s_x^2 + \frac{1}{n-1}\sum(x_i - \bar{x})\varepsilon_i}$$

$$E\left(\beta_1 s_x^2 + \frac{1}{n-1}\sum (x_i - \bar{x})\varepsilon_i\right) = \boldsymbol{\beta_1 s_x^2} = \boldsymbol{E(cov(x,Y))}$$

$$\boldsymbol{E(y)} = \frac{1}{n}E\left(\sum y_i\right) = \frac{1}{n}E\left(\sum \beta_0 + \beta_1 x_i + \varepsilon_i\right) = \boldsymbol{\beta_0 + \beta_1 \bar{x}}$$

Now, let's assume that the standard addition is independently applied $j = 1, \dots, n_y$ different times, i.e. each time $n = n_r n_e$ observations are generated. Then n_y different mean values $\bar{y}_{1j}$ for the unspiked measurements can be calculated:

$$\bar{\boldsymbol{y}}_{\boldsymbol{1j}} = \frac{1}{n_r}\sum_{k=1}^{n_r} y_{k1j} = \beta_0 + \frac{1}{n_r}\sum_{k=1}^{n_r}\varepsilon_{k1j} \Rightarrow \bar{\boldsymbol{y}}_{\boldsymbol{1j}} - \boldsymbol{\beta_0} = \frac{\boldsymbol{1}}{\boldsymbol{n_r}}\sum_{\boldsymbol{k=1}}^{\boldsymbol{n_r}}\boldsymbol{\varepsilon_{k1j}}$$

$$\Downarrow$$

$$\boldsymbol{cov(\bar{y}_1, cov(x,Y))} = \frac{1}{n_y}\sum_{j=1}^{n_y}(\bar{y}_{1j} - \beta_0)\left(\frac{1}{n}\sum_{i=1}^{n}(x_i - \bar{x})(y_{ij} - \beta_0 - \beta_1\bar{x}) - \beta_1 s_x^2\right)$$

$$= \frac{1}{n_y}\sum(\bar{y}_{1j} - \beta_0)\left(\frac{1}{n}\sum(x_i - \bar{x})(\beta_0 + \beta_1 x_i + \varepsilon_{ij} - \beta_0 - \beta_1\bar{x}) - \beta_1 s_x^2\right)$$

$$= \frac{1}{n_y}\sum(\bar{y}_{1j} - \beta_0)\left(\frac{1}{n}\sum(x_i - \bar{x})(\beta_1(x_i - \bar{x}) + \varepsilon_{ij}) - \beta_1 s_x^2\right)$$

$$= \frac{1}{n_y}\sum(\bar{y}_{1j} - \beta_0)\left(\frac{1}{n}\sum\left(\beta_1(x_i - \bar{x})^2 + \varepsilon_{ij}(x_i - \bar{x})\right) - \beta_1 s_x^2\right)$$

$$= \frac{1}{n_y}\sum\left(\frac{1}{n_r}\sum_{k=1}^{n_r}\varepsilon_{k1j}\right)\cdot\left(\frac{1}{n}\sum\left(\beta_1(x_i - \bar{x})^2 + \varepsilon_{ij}(x_i - \bar{x})\right) - \beta_1 s_x^2\right)$$

$$= \frac{1}{n_y}\sum_{j=1}^{n_y}\frac{1}{n}\sum_{i=1}^{n}\left(\left(\frac{1}{n_r}\sum_{k=1}^{n_r}\varepsilon_{k1j}\right)\beta_1(x_i - \bar{x})^2 + \left(\frac{1}{n_r}\sum_{k=1}^{n_r}\varepsilon_{k1j}\right)\varepsilon_{kij}(x_i - \bar{x}) - \left(\frac{1}{n_r}\sum_{k=1}^{n_r}\varepsilon_{k1j}\right)\beta_1 s_x^2\right)$$

$$= (\dots)$$

$$\Rightarrow E(\dots) = E\left(\frac{1}{n_y}\sum_{j=1}^{n_y}\frac{1}{n}\sum_{i=1}^{n}\left(\frac{1}{n_r}\sum_{k=1}^{n_r}\varepsilon_{k1j}\right)\varepsilon_{kij}(x_i - \bar{x})\right)$$

$$= E\left(\frac{1}{n_y}\sum_{j=1}^{n_y}\frac{1}{n}\frac{1}{n_r}\sum_{i=1}^{n_r}\varepsilon_{k1j}^2(x_1 - \bar{x})\right)$$

$$= \frac{1}{n_y}\sum_{j=1}^{n_y}\frac{1}{n}\frac{1}{n_r}\sum_{i=1}^{n_r}E\left(\varepsilon_{k1j}^2(x_1 - \bar{x})\right)$$

$$= \frac{\boldsymbol{1}}{\boldsymbol{n}}\boldsymbol{\sigma^2(x_1 - \bar{x})} = -\frac{\boldsymbol{1}}{\boldsymbol{n}}\boldsymbol{\sigma^2\bar{x}} < \boldsymbol{0}$$

For

$$\sigma^2_{\hat{C}_0^i} \approx \left(\left(\frac{\sigma_{\hat{\beta}_0^i}}{\beta_0}\right)^2 + \left(\frac{\sigma_{\hat{\beta}_1}}{\beta_1}\right)^2 - \frac{2cov(\hat{\beta}_0^i, \hat{\beta}_1)}{\beta_0\beta_1} \right) C_0^2$$

we have therefore

$$\sigma^2_{\hat{\boldsymbol{\beta}}_0^i} = 4var(\bar{y}_1) + var(\hat{\beta}_0) - 4cov(\bar{y}_1, \hat{\beta}_0)$$

$$= \frac{4\sigma^2}{n_r} + var(\hat{\beta}_0) - 4\left(cov(\bar{y}_1, \bar{Y}) - \bar{x}cov(\bar{y}_1, \hat{\beta}_1)\right)$$

$$= \frac{4\sigma^2}{n_r} + var(\hat{\beta}_0) - 4\left(\frac{1}{n}\sigma^2 - \frac{\bar{x}}{s_x^2} cov(\bar{y}_1\,, cov(x, Y))\right)$$

$$= \frac{4\sigma^2}{n_r} + var(\hat{\beta}_0) - 4\left(\frac{1}{n}\sigma^2 - \frac{\bar{x}}{s_x^2}\frac{1}{n}\sigma^2(x_1 - \bar{x})\right)$$

$$= \frac{4\sigma^2}{n_r} + var(\hat{\beta}_0) - \frac{4\sigma^2}{n}\left(1 - \frac{\bar{x}}{s_x^2}(x_1 - \bar{x})\right)$$

$$= \frac{4\sigma^2}{n_r} + var(\hat{\beta}_0) - \frac{4\sigma^2}{n}\left(1 - \frac{1}{s_x^2}(x_1\bar{x} - \bar{x}^2)\right)$$

and, since $x_1 = 0$

$$\boldsymbol{\sigma^2_{\hat{\beta}_0^i} = \frac{4\sigma^2}{n_r} + var(\hat{\beta}_0) - \frac{4\sigma^2}{n}\left(1 + \frac{\bar{x}^2}{s_x^2}\right) = var(\hat{\beta}_0) + 4\sigma^2\left(\frac{1}{n_r} - \frac{1}{n}\left(1 + \frac{\bar{x}^2}{s_x^2}\right)\right)}$$

Since

$$1 + \frac{\bar{x}^2}{s_x^2} > 0$$

it is not easy to see that $\sigma^2_{\hat{\beta}_0^i} > \sigma^2_{\hat{\beta}_0^e}$, but has to be proven:

With

$$n_e = \frac{n}{n_r} \text{ and } (x_1 - \bar{x})^2 = (x_{n_e} - \bar{x})^2 = \bar{x}^2$$

we get, that

$$\frac{1}{n} + \frac{\bar{x}^2}{(n-1)s_x^2} = \frac{1}{n} + \frac{\bar{x}^2}{2n_r\bar{x}^2 + n_r(x_2 - \bar{x})^2 + \dots + n_r\left(x_{n_e - 1} - \bar{x}\right)^2} < \frac{1}{n} + \frac{1}{2n_r} =$$

$$\frac{1}{n_r}\left(\frac{1}{n_e} + \frac{1}{2}\right) \le |n_e \ge 2| \le \frac{1}{n_r}$$

and therefore

$$(\mathbf{1}) \quad \boldsymbol{var(\hat{\beta}_0^i)} = \frac{4\sigma^2}{n_r} + var(\hat{\beta}_0) - 4\sigma^2\left(\frac{1}{n} + \frac{\bar{x}^2}{ns_x^2}\right) > \boldsymbol{var(\hat{\beta}_0)}$$

$$\boldsymbol{cov}\left(\widehat{\boldsymbol{\beta}}_{\mathbf{0}}^{\boldsymbol{i}}, \widehat{\boldsymbol{\beta}}_{\mathbf{1}}\right) = 2cov\left(\bar{y}_1, \hat{\beta}_1\right) - cov(\hat{\beta}_0, \hat{\beta}_1)$$

$$= \frac{2}{s_x^2} cov\left(\bar{y}_1, cov(x, Y)\right) - cov(\hat{\beta}_0, \hat{\beta}_1)$$

$$= -\frac{2}{ns_x^2}\sigma^2\bar{x} - cov(\hat{\beta}_0, \hat{\beta}_1)$$

$$= -\frac{2}{ns_x^2}\sigma^2\bar{x} - \frac{-\sum_{i=1}^n x_i}{\sqrt{n\sum_{i=1}^n x_i^2}}\,\sigma\left(\frac{\sum_{i=1}^n x_i^2}{n\sum_{i=1}^n (x_i-\bar{x})^2}\right)^{\frac{1}{2}}\sigma\left(\frac{1}{\sum_{i=1}^n (x_i-\bar{x})^2}\right)^{\frac{1}{2}}$$

$$= -\frac{2}{ns_x^2}\sigma^2\bar{x} + \frac{1}{n}\sigma^2\sum_{i=1}^n x_i\left(\frac{1}{\sum_{i=1}^n (x_i-\bar{x})^2}\right)^{\frac{1}{2}}\left(\frac{1}{\sum_{i=1}^n (x_i-\bar{x})^2}\right)^{\frac{1}{2}}$$

$$= -\frac{2}{ns_x^2}\sigma^2\bar{x} + \frac{\sigma^2\bar{x}}{ns_x^2} = -\frac{\boldsymbol{\sigma^2\bar{x}}}{\boldsymbol{ns_x^2}} = \boldsymbol{cov}\left(\widehat{\boldsymbol{\beta}}_{\mathbf{0}}, \widehat{\boldsymbol{\beta}}_{\mathbf{1}}\right) \quad (\mathbf{2})$$

$$(\mathbf{1}) + (\mathbf{2}) \quad \Rightarrow \quad \boldsymbol{\sigma}^2_{\hat{C}_0^e} < \boldsymbol{\sigma}^2_{\hat{C}_0^i}$$

Variance Normalization Approach

Applying the method of error propagation to approximate the variance of the slope estimator yields:

$$i = 1{:}\quad \frac{\partial}{\partial y_{11}}\left(\sum_{i=1}^{n_e} y_{1i}^n x_i\right) = \frac{\partial}{\partial y_{11}}\left(\frac{1}{y_{11}}\sum_{i>1}^{n_e} y_{1i}x_i + x_1\right) = -\frac{1}{(y_{11})^2}\sum_{i>1}^{n_e} y_{1i}x_i$$

$$i > 1{:}\quad \frac{\partial}{\partial y_{1i}}\left(\sum_{i=1}^{n_e} y_{1i}^n x_i\right) = \frac{x_i}{y_{11}}$$

i.e., with $s_x^2 = \sum_{i=1}^n x_i^2 = n_s\sum_{i=1}^{n_r} x_i^2$, we get

$$i = 1, j = 1, \ldots, n_r:\quad \frac{\partial\hat{\beta}}{\partial y_{j1}} = \frac{1}{\sum_{i=1}^n x_i^2}\left(-\frac{1}{\left(y_{j1}\right)^2}\sum_{i>1}^{n_e} y_{ji}x_i\right)$$

$$i = 2, \ldots, n_e, j = 1, \ldots, n_r:\quad \frac{\partial\hat{\beta}}{\partial y_{ij}} = \frac{1}{\sum_{i=1}^n x_i^2}\left(\frac{x_i}{y_{j1}}\right)$$

and therefore, by denoting

$$y_{1i0} = E(Y|x_{1i}), \qquad i = 1, \ldots, n_e$$

the formula for the approximate variance of $\hat{\beta}$, $var(\hat{\beta})$, is

$$var(\hat{\beta}) = \frac{n_r \sigma^2}{\left(\sum_{i=1}^{n} x_i^2\right)^2} \left(\left(-\frac{1}{(y_{110})^2} \sum_{i>1}^{n_e} y_{1i0} x_i \right)^2 + \left(\frac{1}{y_{110}}\right)^2 \sum_{i>1}^{n_e} x_i^2 \right)$$

and that for $var(\hat{C}_0)$ is accordingly given by $\left(\hat{C}_0 = 1/\hat{\beta}\right)$:

$$\frac{\partial \hat{C}_0}{\partial \hat{\beta}} = -\frac{1}{\hat{\beta}^2} \Longrightarrow var(\hat{C}_0) \approx \frac{1}{\beta^4} var(\hat{\beta}) = \left(\frac{\beta_0}{\beta_1}\right)^4 var(\hat{\beta})$$

and since $y_{110} = \beta_0$

$$\left(\frac{\beta_0}{\beta_1}\right)^4 var(\hat{\beta}) = \left(\frac{\beta_0}{\beta_1}\right)^4 \frac{n_r \sigma^2}{\left(\sum_{i=1}^{n} x_i^2\right)^2} \left(\left(-\frac{1}{(y_{110})^2} \sum_{i>1}^{n_e} y_{1i0} x_i \right)^2 + \left(\frac{1}{y_{110}}\right)^2 \sum_{i>1}^{n_e} x_i^2 \right) =$$

$$\left(\frac{\beta_0}{\beta_1}\right)^4 \frac{n_r \sigma^2}{\left(\sum_{i=1}^{n} x_i^2\right)^2} \left(\left(-\frac{1}{(\beta_0)^2} \sum_{i>1}^{n_e} y_{1i0} x_i \right)^2 + \left(\frac{1}{\beta_0}\right)^2 \sum_{i>1}^{n_e} x_i^2 \right) =$$

$$\frac{\beta_0^2 n_r \sigma^2}{\left(\beta_1^2 \sum_{i=1}^{n} x_i^2\right)^2} \left(\left(\frac{1}{\beta_0}\right)^2 \left(\sum_{i>1}^{n_e} y_{1i0} x_i \right)^2 + \sum_{i>1}^{n_e} x_i^2 \right) =$$

$$\frac{C_0^2 n_r \sigma^2}{\left(\beta_1 \sum_{i=1}^{n} x_i^2\right)^2} \left(\left(\frac{1}{\beta_0}\right)^2 \left(\sum_{i>1}^{n_e} y_{1i0} x_i \right)^2 + \sum_{i>1}^{n_e} x_i^2 \right) = \left| \frac{C_0^2}{\beta_1^2} = \frac{\beta_0^2 C_0^2}{\beta_0^2 \beta_1^2} = \frac{C_0^4}{\beta_0^2} \right| =$$

$$\frac{C_0^4 n_r \sigma^2}{\left(\beta_0 \sum_{i=1}^{n} x_i^2\right)^2} \left(\left(\frac{1}{\beta_0}\right)^2 \left(\sum_{i>1}^{n_e} y_{1i0} x_i \right)^2 + \sum_{i>1}^{n_e} x_i^2 \right)$$
